# Perspectives on the DNR decision process: A survey of nurses and physicians in hematology and oncology

**DOI:** 10.1371/journal.pone.0206550

**Published:** 2018-11-21

**Authors:** Mona Pettersson, Anna T. Höglund, Mariann Hedström

**Affiliations:** Department of Public Health and Caring Sciences, Uppsala University, Uppsala, Sweden; Universiteit van Amsterdam, NETHERLANDS

## Abstract

**Introduction:**

In cancer care, do-not-resuscitate (DNR) decisions are made frequently; i.e., decisions not to start the heart in the event of a cardiac arrest. A DNR decision can be a complex process involving nurses and physicians with a wide variety of experiences and perspectives. Previous studies have shown different perceptions of the DNR decision process among nurses and physicians, e.g. concerning patient involvement and information. DNR decisions have also been reported to be unclear and documentation inconsistent.

**Objective:**

The aim was to investigate how important and how likely to happen nurses and physicians considered various aspects of the DNR decision process, regarding participation, information and documentation, as well as which attributes they found most important in relation to DNR decisions.

**Methods:**

A descriptive correlational study using a web survey was conducted, including 132 nurses and 84 physicians working in hematology and oncology.

**Results:**

Almost half of the respondents reported it not likely that the patient would be involved in the decision on DNR, and 21% found it unimportant to inform patients of the DNR decision. Further, 57% reported that providing information to the patient was important, but only 21% stated that this was likely to happen. There were differences between nurses and physicians, especially regarding participation by and information to patients and relatives. The attributes deemed most important for both nurses and physicians pertained more to medical viewpoints than to ethical values, but a difference was found, as nurses chose patient autonomy as the most important value, while physicians rated non-maleficence as the most important value in relation to DNR decisions.

**Conclusion:**

Nurses and physicians need to be able to talk openly about their different perspectives on DNR decisions, so that they can develop a deeper understanding of the decisions, especially in cases where they disagree. They should also be aware that what they think is important is not always likely to happen. The organization needs to support such discussions through providing an environment that allows ethical discussions on regular basis. Patients and relatives will also benefit from receiving the same information from all caregivers.

## Introduction

In cancer care, do-not-resuscitate (DNR) decisions are made frequently; i.e., decisions not to start the heart in the event of a cardiac arrest. DNR decisions are made when a patient has declined resuscitation, a poor prognosis, or if it is considered that the patient will not survive cardiopulmonary resuscitation (CPR) with sufficient quality of life [1]. The WHO definition of Quality of Life refers to the individual's subjective perception of where and how they live, related to goals, expectations, standards and concerns [2]. DNR orders involve refraining from basic CPR, chest compressions with or without simultaneous ventilation, and advanced CPR that includes defibrillation and drugs [3]. If a patient does not have a DNR order, CPR must start within 60 seconds, and defibrillation within three minutes, according to Swedish guidelines [3].

According to Swedish law, the physician responsible for the patient shall decide on DNR [4]. This should be done in consultation with other certified professionals (physicians or nurses), preferably after discussion with the patient, but the final decision is always the physician’s. [4]. The Patient Act further strengthens the patient’s right to participate in his/her care and the right to receive information [5]. If the information cannot be given to the patient, it should, if possible, be given to a relative [5]. Decisions on DNR should be documented in the patient’s medical record together with information on the reasons why the decision was made and who was involved in making the decision. Information on whether or not the decision was made after discussion with the patient, how the patient and relatives were informed of the decision and what attitude toward the decision they expressed, should also be documented [4].

DNR was first described in the early1970s, and already in 1974, the American Medical Association proposed that the decision should be documented in patients’ medical records and communicated to staff members, who provide care for the patient [6]. Studies have shown that DNR orders can be unclear [7, 8], and that documentation on DNR can be inconsistent and varying [7, 9, 10]. Findings also show that DNR decisions are often made late in patients’ care [7, 11–13]. According to Duplan and Pirret [14], the DNR documentation seldom reveals whether the patient and/or family have been informed. When proper documentation is missing, patients can risk receiving unwanted CPR [15, 16].

Studies of nurses and physicians have shown that representatives of these professions may have different approaches to DNR decisions. While physicians are responsible for the decision and have a deeper medical knowledge, nurses spend more time with the patient, providing bedside nursing and medical care during several hours of their work day, and therefore often initiate DNR discussions [7, 17, 18]. One study found that physicians made DNR decisions in consultation with physician colleagues, but without involving nurses or patients [19]. Other studies have shown that nurses preferred DNR decisions to be made after discussions with patients [7, 20–22]. Nurses have also expressed the need for clear and well-documented DNR orders and informed patients and relatives, in order to provide good nursing care, such as extra support to the families, helping the patient to a peaceful death and avoiding unwanted CPR [7, 15]. Pfeil et al. [21] found that physicians could have a proactive role in which they informed all patients of a DNR decision, or a passive role, in which they waited for the patient to initiate the discussion.

Barriers to making clear DNR decisions could be the physician’s desire not to cause worry in the patient [18] and not to take away the patient’s hope [18, 23], but also lack of experience and feelings of discomfort [18, 24]. Timing of the discussion [25, 26] and the risk of making the wrong decision [25] have also been mentioned as barriers. Studies have also revealed that physicians do not always discuss DNR decisions with their patients [10, 20].

Ethical decisions, such as DNR decisions, can be difficult and lead to conflicts of interests for the involved parties. Examples of such conflicts of interest are disagreements in the team about if there should be a DNR decision or not, and if the patient and/or relatives should be informed or not [7]. Physicians have also reported experiencing ethical conflicts when regulations require information to patients about the decision, but they consider it being against the patient’s best interest [27].

In sum, a DNR decision can be a complex process, involving nurses and physicians with different experiences and perspectives. Hence, the aim of this study was to investigate 1) how important, and how likely to happen, nurses and physicians working in oncology and hematology considered various aspects of the DNR decision process; 2) which attributes they found most important in relation to DNR decisions; and 3) whether there were differences in ratings between nurses and physicians.

## Materials and methods

### Design

A descriptive correlational study, using a web survey presenting a vignette (a brief history about a fictitious patient case) as a starting point [28], was performed.

### Procedure and participants

**Fig 1:**
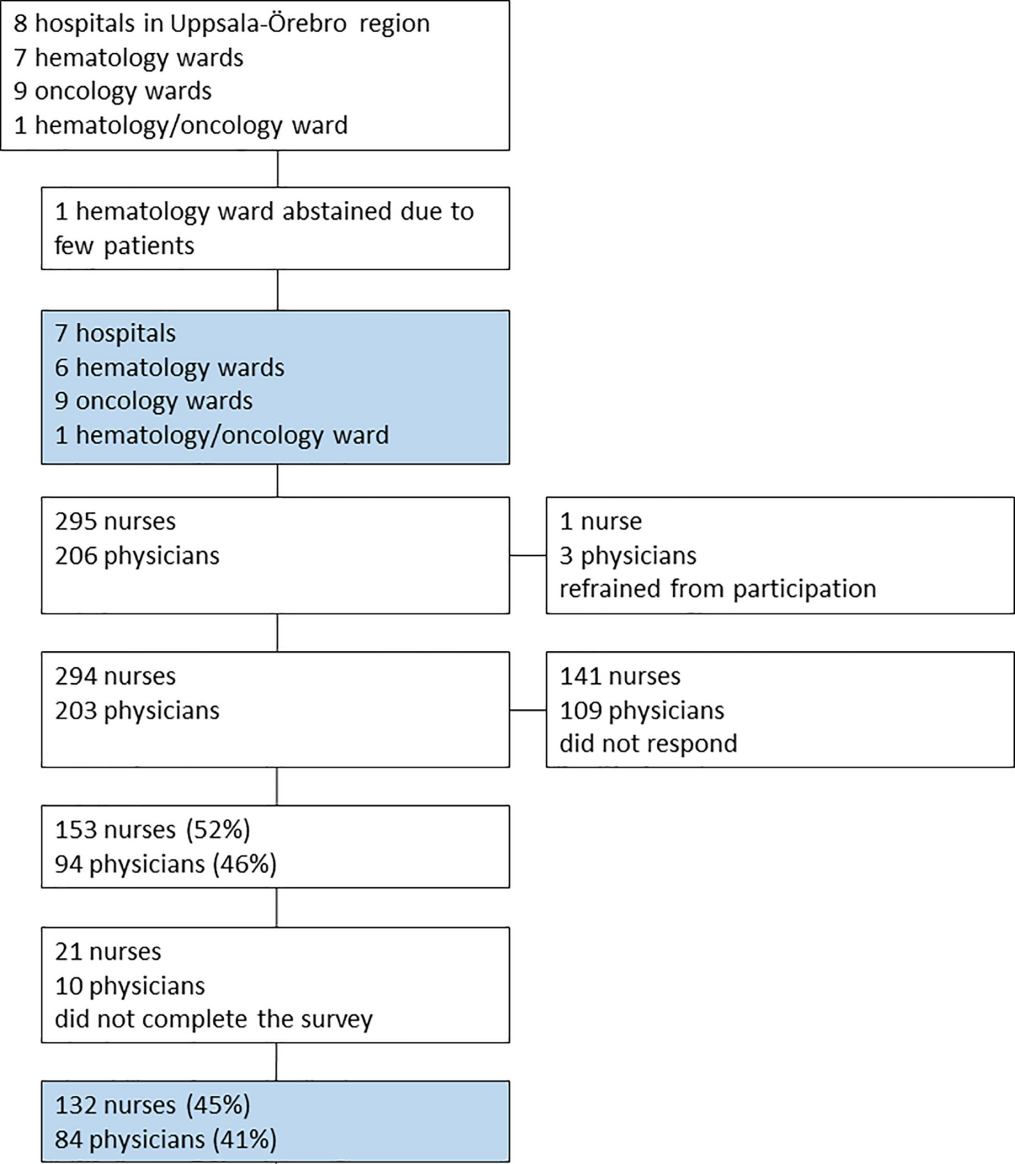
Enrollment of participants.

Heads of departments at seven hospitals, in total 16 hematology and oncology wards, were approached and accepted participation. Among these, six were hematology wards, nine were oncology wards and one ward treated both hematology and oncology patients. At an eighth hospital, the head of the department at a hematology ward declined to participate due to the department handling very few patients with the required diagnoses. Enrollment in the study, including external dropouts, is presented in Fig 1.

Data were collected from February to October, 2017. Email addresses of physicians and nurses who had worked for at least 6 months in hematology and oncology wards were provided by a ward manager or other coordinators (physicians and administrators). Information about the background and purpose of the study and a link to the web survey were sent to the presumptive participants via email. As the web survey was anonymous, all presumptive participants received at least two reminders. Several wards received a personal visit from the first author, who provided brief information about the study and the project of which the study is a part. During the visit, paper surveys and prepaid envelopes were distributed to those who preferred to complete the survey that way.

The web survey was sent to 295 nurses and 206 physicians. The response rate was 132 (45%) for nurses and 84 (41%) for physicians, a total of 216 (43%) (Fig 1). Twenty-one nurses (no physicians) responded via paper surveys. Of the participants, 63 nurses and 25 physicians (n = 88) worked in hematology (41%), and 69 nurses and 59 physicians (n = 128) worked in oncology (59%). The majority of the respondents were female (nurses 96%, physicians 57%). Physicians had a higher mean age (47 years) than nurses (37 years) and had worked longer in the profession (mean 18 years vs 10 years). More physicians (76%) than nurses (14%) had specialist training. Characteristics of participants are presented in Table 1.

**Table 1:** Background variables for the sample (n = 216).

		*Respondents (n)*	Total sample	Working in hematology	Working in oncology
**Nurses**	n (%)		132	63 (48)	69 (52)
Age	M (range)	*128*	37 (22–66)	36 (23–64)	39 (22–66)
Gender	F/M (%)	*131*	126/5 (96/4)	59/3 (95/5)	67/2 (97/3)
Years in profession	M (range)	*108*	10 (0.5–44)	10 (0.5–38)	10 (0.5–45)
Specialist training	n (%)	*NA*	18 (14)	7 (11)	11 (16)
Years in oncology/hematology	M (range)	*108*	NA	7 (0.5–33)	8 (0–31)
**Physicians**	n (%)	*84*	84	25 (30)	59 (70)
Age	M (range)	*82*	47 (27–67)	49 (30–67)	46 (27–65)
Gender	F/M (%)	*84*	48/36 (57/43)	12/13 (48/52)	36/23 (61/39)
Years in profession	M (range)	*84*	18 (1–41)	20 (6–39)	16 (1–39)
Specialist training	n (%)	*NA*	64 (76)	24 (96)	40 (68)
Years in oncology/hematology	M (range)	*80*	NA	12 (1–31)	11 (0.5–33)

### Data collection

The study-specific web survey was developed by the authors, based on aspects of DNR decisions that are regulated in Swedish law, e.g., participation, information and documentation [4, 5] and on findings from previous studies reporting problems with those aspects [7, 16–18, 27]. The web survey was designed using the Survey Monkey survey tool. The following sections were included: first, respondents were asked to fill in background data (see Table 1) and to choose a specialty, oncology or hematology. Based on this choice, the respondents were directed to a short vignette, presenting either an oncology or a hematology fictitious patient case (Table 2). The patient cases were comparable in the patient’s name, age, gender, history of illness and progress of illness, and presented an illustration of the conditions for a decision on DNR.

**Table 2:** Vignettes.

**Hematology** Erik Larsson, 75 years. Married, two adult children Previous Diseases: Chronic Obstructive Pulmonary Disease (COPD) diagnosed 5 years ago. Smoking cessation and local treatment. Myocardial infarction 3 years ago. Angina pectoris occasionally.	**Oncology** Erik Larsson, 75 years. Married, two adult children Previous Diseases: Chronic Obstructive Pulmonary Disease (COPD) diagnosed 5 years ago. Smoking cessation and local treatment. Myocardial infarction 3 years ago. Angina pectoris occasionally.
Diagnosed with multiple myeloma six years ago. Treatment was discussed and autologous stem cell transplant was not chosen due to age and concurrent diseases. Received custom first line treatment according to national care program. Transfusions initially for anemia and thrombocytopenia. Plateau phase was reached after 9 months. Relapse after four years and new treatment began with second-line treatment according to the care program. Back pain was treated with long-acting morphine. Two spontaneous fractures (arm and shoulder) were irradiated with good results and plateau phase was reached again after 11 months of treatment. During treatment, repeated pulmonary infections. COPD follow-up showed 50% lung capacity. Six months after the second treatment, Erik returns to the emergency room with pneumonia, increasing cough and breathing difficulties. He is exhausted, but awake and cognitively unaffected. Erik is admitted to your ward. Relatives visit every day, and they are very concerned about Erik's worsening condition. The back pain is worse and blood tests show anemia and increased infection rates. Discussion about a DNR decision is pertinent.	Heredity for prostate cancer (father and brother). High PSA values were detected through screening, leading to start of oral hormone treatment. After one year, PSA increased further, and transition to hormone injections started, given every third month. Irradiation toward the prostate was given a total of 35 times. Two years later, lymph glands were detected in the lower abdomen. These were successfully irradiated, in a total of 25 events. Subsequent problems with swollen legs and pain, which were well treated with compression stocking and pain relief. After another two years, new relapse treated with chemotherapy. A total of 5 occasions were planned, but after the fourth occasion, Erik had a bone marrow failure. Pneumonia was also detected. Treatment started with antibiotics and transfusion support during a two-week placement. Pain increased in the back and suspicion of skeletal metastases were confirmed in a skeletal scintigraphy. Follow-up of COPD showed 50% lung capacity. A month later, Erik suffers from a new pneumonia, with increasing cough and breathing difficulties. He is exhausted, but awake and cognitively unaffected. Erik is admitted to your ward. Relatives visit every day, and they are very concerned about Erik's worsening condition. The PSA value has risen again since the chemotherapy treatment ended. Discussion about a DNR decision is pertinent.
*Definition of DNR in this document*: *a decision not to perform basic CPR (chest compression and assisted breathing) or advanced CPR (defibrillator or medical drugs)*.	*Definition of DNR in this document*: *a decision not to perform basic CPR (chest compression and assisted breathing) or advanced CPR (defibrillator or medical drugs)*.

Subsequently there were questions in which respondents estimated, on a 6-step Likert scale from 1 (not important/likely) to 6 (very important/likely), how *important* an aspect of the DNR decision process was and in the next question how *likely* this was to happen at the ward. The participants were asked to answer the questions regarding importance according to the information in the vignette, and the questions regarding likelihood according to their experience from their present workplace.

The questions were phrased as follows:

*How important/How likely* do you think it is that a DNR decision is taken after discussion with the patient/relative?
*How important/How likely* do you think it is that a DNR decision is taken in consultation with another physician/nurse?
*How important/How likely* do you think it is that the patient/relative/care team is informed about the DNR decision?
*How important/How likely* do you think it is that a DNR decision is clearly documented?

As previous studies have shown that DNR decisions are of high ethical relevance to nurses and physicians [7, 27], the respondents were also asked to choose the three (out of eight) most important attributes in relation to DNR decisions. The list of choices included the following ethically relevant attributes: survival and quality of life after resuscitation; medical prognosis; the right to a peaceful death; patient autonomy; principle of non-maleficence; principle of beneficence; patient age; and opinions of relatives. The list was based on the outcome of interviews about ethical competence with nurses and physicians [27]. The attributes are not considered mutually exclusive by the authors.

### Considerations of validity and reliability

Content validity was supported by the fact that the items chosen for the survey were based on aspects of DNR decisions regulated in Swedish law and guidelines [1, 4, 5] and on qualitative interviews with both nurses and physicians [7, 27]. Using a fictitious patient case, a vignette, gave the respondents the setting for a decision which they were about to make/participate in, which was an opportunity to collect respondents’ perceptions of such a situation [28]. The two patient cases were reviewed and edited in consultation with a physician who is Professor of hematology. Two nurses working in hematology reviewed the questions for the survey, and small adjustments were made. The final web survey was tested on 15 nurses who teach at the nursing program at Uppsala University. They did not report difficulties in understanding the questions or handling the web tool for the survey, but had two comments, which led to the rephrasing of one question, and a slightly modified design.

### Analysis

Data analyses were made in Statistical Packages for the Social Sciences [SPSS], version 24 (S1 File). Data were presented descriptively using numbers, percent, and measures for central tendency and range. Further, answers to the questions about importance and likelihood were grouped into “unimportant/unlikely” defined as scoring 1–2 on the Likert scale, “neither important/likely nor unimportant/unlikely” (3–4) and “important/likely” (5–6). The statistical analyses were made with Mann-Whitney U tests for ordinal data and Chi Square tests for categorical data. Alpha was set after Bonferroni correction (0.05/16 = 0.003 for important/likely ratings and 0.05/8 = 0.006 for attributes).

In response to the question about the three most important attributes in relation to DNR decisions, ten participants made no choice, and one participant made four. These answers were discarded from the analysis. Four participants made two choices and three participants made one choice, and these were included in the analysis.

### Ethical considerations

Approval for the study was sought from the Regional Ethics Review Board, which responded that no ethical approval was required for the study (Reg. no. 2016/484). The study was conducted according to national and international guidelines and regulation for empirical research [29, 30].

The department heads of the respective clinics in each hospital were contacted about the study and gave permission to request email addresses of physicians and nurses. Information about the study was sent to all nurses and physicians along with a link to the web survey. The information stated that participation was voluntary, that data could not be connected to any person, department or hospital, and that data would be reported on group levels (nurses/physicians and hematology/oncology). Further, information was given that reminders would be sent to all participants, and identification of individuals who had already answered the questionnaire would therefore not be possible. By responding to the survey, participants agreed to the results being published in a scientific journal. No questions considered sensitive or harmful were asked in the survey.

## Results

### Importance and likelihood—Aspects of the DNR decision process

Table 3 presents the respondents’ answers to how important, and how likely to happen at their workplace, they considered various aspects of the DNR decision process in relation to the presented patient case. The largest proportion of respondents thought it was important for a DNR decision to be taken after discussion with the patient and his relatives, and in consultation with nurses, but unlikely that this would happen at their place of work. The majority thought it important for the patient and his relatives to be informed of the decision, but not as many thought it was likely to happen. The vast majority thought it both important, and likely to happen, that the care team would be informed of the decision, and that the decision would be clearly documented (Table 3).

**Table 3:** Ratings of how *important* an aspect of the DNR decision process is and how *likely* it is that this happens at the ward.

		*n*	Md; Range	Unimportant or unlikely	Neither important nor unimportant or likely nor unlikely	Important or likely
**Discussion with patient**	Important	*216*	5; 1–6	34 (16%)	66 (30%)	116 (54%)
** **	Likely	*216*	3; 1–6	98 (45%)	82 (38%)	36 (17%)
**Discussion with relatives**	Important	*210*	5; 1–6	42 (20%)	63 (30%)	105 (50%)
** **	Likely	*212*	3, 1–6	90 (42%)	85 (40%)	37 (18%)
**Consultation with other physicians**	Important	*212*	4; 1–6	48 (22%)	75 (36%)	89 (42%)
** **	Likely	*213*	4; 1–6	61 (29%)	86 (40%)	66 (31%)
**Consultation with nurses**	Important	*208*	4; 1–6	32 (15%)	76 (37%)	100 (48%)
** **	Likely	*212*	3; 1–6	85 (40%)	67 (32%)	60 (28%)
**Information to patient**	Important	*206*	5; 1–6	44 (21%)	46 (22%)	116 (57%)
** **	Likely	*205*	3; 1–6	84 (41%)	77 (38%)	44 (21%)
**Information to relatives**	Important	*203*	5; 1–6	24 (12%)	40 (19%)	139 (69%)
** **	Likely	*203*	4; 1–6	55 (27%)	76 (37%)	72 (36%)
**Information to care team**	Important	*204*	6; 1–6	1 (1%)	5 (2%)	198 (97%)
** **	Likely	*204*	6; 1–6	9 (5%)	22 (11%)	173 (84%)
**Distinct documentation**	Important	*205*	6; 1–6	1 (.1%)	1 (1%)	203 (98%)
** **	Likely	*206*	6; 2–6	6 (3%)	20 (10%)	180 (87%)

Median and range for all respondents and percent of respondents answering unimportant or unlikely (scoring 1–2 on the Likert scale), neither important nor unimportant or likely nor unlikely (3–4) and important or likely (5–6) (n = 216).

### Importance and likelihood–nurses and physicians

Nurses rated the importance of making the DNR decision after discussion with the patient and relatives, as well as the importance of informing the patient and relatives of the decision, higher than physicians did. Clear documentation of the decision was rated less likely to happen by nurses than by physicians (Table 4).

**Table 4:** Ratings of how *important* an aspect of the DNR decision process is and how *likely* it is that this happens at the ward. Differences between nurses (n = 132) and physicians (n = 84).

		Nurse		Physician		z	p
		*n*	M;Md;Range	*n*	M;Md;Range		
**Discussion with patient**	Important	*132*	4.92; 5; 1–6	*84*	3.51; 3.5; 1–6	-6.43	**.000**
	Likely	*132*	3.05; 3; 1–6	*84*	2.82; 2; 1–6	-1.31	.190
**Discussion with relatives**	Important	*126*	4.64; 5; 1–6	*84*	3.61; 4; 1–6	-4.66	**.000**
	Likely	*128*	3.13; 3; 1–6	*84*	2.99; 3; 1–6	-.79	.429
**Consultation with other physicians**	Important	*129*	3.91; 4; 1–6	*83*	3.96; 4; 1–6	-.11	.911
	Likely	*130*	3.42, 3; 1–6	*83*	3.82; 4; 1–6	-1.85	.064
**Consultation with nurses**	Important	*127*	4.45; 5; 1–6	*81*	3.96; 4; 1–6	-2.26	.024
	Likely	*131*	3.04; 3; 1–6	*81*	3.70, 4; 1–6	-2.79	.005
**Information to** **patient**	Important	*125*	4.97; 5; 1–6	*81*	3.26; 3; 1–6	-7.10	**.000**
	Likely	*124*	3.40; 3; 1–6	*81*	2.86; 2; 1–6	-2.45	.014
**Information to** **relatives**	Important	*125*	5.32, 6; 1–6	*78*	3.96; 4; 1–6	6.94	**.000**
	Likely	*125*	3.86; 4; 1–6	*78*	3.44; 3; 1–6	-1.96	.050
**Information to care team**	Important	*125*	5.94; 6; 3–6	*79*	5.75; 6; 1–6	-2.58	.010
	Likely	*124*	5.42; 6; 2–6	*80*	5.28; 6; 1–6	-1.52	.128
**Distinct documentation**	Important	*124*	5.98; 6; 4–6	*81*	5.86; 6; 1–6	-2.81	.005
	Likely	*125*	5.18; 6; 2–6	*81*	5.68; 6; 2–6	-3.67	**.000**

### Important attributes in relation to DNR decisions

Participants were asked to choose the three (out of eight) most important attributes in relation to DNR decisions. The top three attributes overall, regardless of profession, were: survival and quality of life after resuscitation; medical prognosis; and patient’s right to a peaceful death. However, nurses chose patient autonomy more often than physicians, while physicians chose non-maleficence to a higher extent than nurses (Table 5).

**Table 5:** Important attributes in relation to DNR decisions.

	Total n = 205	Nurses n = 124	Physicians n = 81	Chi^2^, p-value
**Survival and quality of life after resuscitation**	178 (87%)	110 (89%)	68 (84%)	.97 .325
**Medical prognosis**	134 (65%)	75 (61%)	59 (73%)	3.30 .069
**Patient’s right to a peaceful death**	118 (58%)	78 (63%)	40 (49%)	3.67 .056
**Patient autonomy**	65 (32%)	51 (41%)	14 (17%)	12.87 **.000**
**Principle of non-maleficence**	62 (30%)	22 (18%)	40 (49%)	23.25 **.000**
**Principle of beneficence**	32 (16%)	17 (14%)	15 (19%)	.86 .354
**Patient age**	13 (6%)	9 (7%)	4 (5%)	.44 .505
**Opinions of relatives**	3 (2%)	2 (2%)	1 (1%)	.05 .825

Numbers and percent of respondents choosing the attribute as one of the three most important in relation to DNR decisions, and analyses of differences between nurses and physicians.

## Discussion

In this study, the introductory vignette gave the premises that this was a cognitively unaffected patient with concerned and engaged relatives. However, 45% of the respondents reported it not likely that the patient would be involved in the decision on DNR and 21% found it unimportant to inform the patient of the DNR decision. Twelve percent found it unimportant to inform relatives. Further, 57% of the respondents reported that providing information to the patient was important, but only 21% stated that this was likely to happen.

These results are striking, as they are not in line with Swedish regulations on DNR [5], which emphasize patient participation and information. On the other hand, the guidelines from the Swedish Society of Medicine et al. [1] state that physicians and nurses have an ethical responsibility to judge if information on DNR decisions can do more harm than good for a patient, which partly can explain these findings. Nevertheless, our results reveal a discrepancy between regulations and clinical practice regarding patient participation and information in DNR decisions in Sweden.

Most respondents thought it both important, and likely to happen, that the care team would be informed of the decision, and that the decision would be clearly documented. This is in contrast to what previous studies have reported on unclear DNR decisions [7, 8] and inconsistent and unclear documentation [7, 10, 16]. The Swedish regulation states that decisions on DNR and other withdrawal of life-sustaining treatment should be documented in the patient’s medical record [4]. In addition, a new guideline for CPR was released in 2013 [1], which may have contributed to enhanced awareness of the requirements for documentation of these decisions.

More respondents rated it important for DNR decisions to be made after discussion with patient or relatives, and asserted that they should be informed of the decision, than rated this likely to happen at their work place. Believing something is of high importance but not likely to happen may lead to situations in which nurses and physicians can experience moral distress. Jameton [31] defined moral distress in nursing as a challenge in which a caregiver’s ethical or moral judgement about care differs from the judgement of those in charge. Young et al. [32] found that nursing staff felt moral distress resulting from powerlessness to do the right thing in situations where they were unable to influence a patient’s end-of-life care. In Prentice et al. [33], nurses and physicians described moral distress when acting against their conscience, when they experienced that patients suffered during their care, or when they were forced to choose between competing ethical principles or unable to act in the patients’ best interest, due to things outside their own control.

Moral distress can also be measured using a moral distress scale as described by Corley et al. [34]. In their scale for nurses, high scores on a Likert scale for a 32-item statement indicated risk for moral distress. Examples of statements were: “Follow the physician’s request not to discuss Code status with patients”, and “Follow the family’s wishes to continue life support even though it is not in the best interest of the patient” [34]. These examples are in line with our results, and indicate that moral distress could occur in relation to DNR decisions.

Nurses rated it more important than physicians that DNR decisions were taken after discussion with the patient and relatives, and that the patient and relatives should be informed of the decision. Nurses also rated this less likely to happen, which according to the reasoning above puts nurses in particular risk of experiencing moral distress. Previous research showed that nurses wanted patients and relatives to be informed of the DNR decision so they could talk openly about it and provide adequate support and care [7]. This might also help the family to accept the situation and prepare the patient for a peaceful death [35].

Among the eight attributes that could be chosen as one of the three most important, some were more related to medical rather than ethical viewpoints. The attributes had been mentioned as important in relation to DNR decisions in an earlier interview study [27]. The three attributes in relation to DNR decisions that were chosen most often were: survival and quality of life after resuscitation; medical prognosis; and patient’s right to a peaceful death. Although all three are possible to interpret in ethical terms, the first two are arguable more related to medical than ethical viewpoints. This could reflect that DNR decisions are primarily seen as medical decisions, made with focus on the patient’s medical condition.

However, both nurses and physicians chose more ethical values in fourth place. Physicians chose non-maleficence as the fourth most common attribute, which is also one of the statements in the physicians Hippocratic Oath [36]. Nurses’ fourth choice was patient autonomy, which could express a belief in the patient’s decision-making capability, and also a wish for patient autonomy in relation to these decisions. This is in line with the nurses’ highly rated importance of making a DNR decision after discussions with patient and relatives, and the importance of informing them of the decision.

Swedish regulations emphasize patient autonomy in relation to DNR decisions [5]. Nurses, who spend long time close to the patient and/or relatives and want them to be informed about a DNR decision to be able to give better care [7], chose patient autonomy as one of the more important attributes in relation to DNR decisions. Physicians, on the other hand, chose non-maleficence as the most important attribute. This is in line with previous research which showed that physicians can provide ethical arguments for not informing patients of DNR decisions, because of the risk to do more harm than good, even if it means deviating from regulations [27].

### Strengths and limitations

Sixteen wards of various sizes were included in the study. Some offered hematology or oncology care exclusively, while some were mixed with another medical or surgical specialty. One ward treated both hematology and oncology patients. Participants were asked to choose the specialty in which they worked, or the most recent one if they worked in both. They were then transferred to a vignette presenting a fictitious patient case in that specialty. The seven hospitals from which participants were recruited were either local hospitals, larger county hospitals or university hospitals, located in cities of various sizes, with both rural and urban catchment areas. This could contribute to the generalizability of the findings.

Ward heads or ward coordinators provided email addresses for all nurses and physicians at the wards who had worked for at least 6 months with oncology and/or hematology patients. All presumptive participants were sent information about the study, including a link to the web survey. In some cases, an auto responder announced parental leave, sick leave or other absence. Some emails bounced due to communication errors with the server. Hence, the response rate could be higher than reported here, but not lower. Nevertheless, the overall response rate of 43% is an area of concern. In addition, not all respondents answered every question, and the response rate is lower in the latter part of the survey. During the personal visits at some of the participating wards, the first author initiated discussions about reasons for and against participating in the survey. Most staff representatives agreed that the topic was important and interesting, and the most frequently mentioned reason for not participating was time shortage.

Baruch and Holtom [37] note that declining figures in response rates are a trend since the 1970s, and response rates as reported in our study are not unusual in recent internet-based healthcare research. Rather, many studies report response rates in the 10–40% range [38–41] and high survey burden and lack of time are commonly discussed as probable reasons for non-response. If this trend continues, future survey research seems to face serious validity threats. Therefore, it may be recommended for department heads in health care to be selective regarding surveys, and only allow those judged to be of high relevance for the department. That would also make it possible to communicate to staff the importance of participating in those surveys.

The Bonferroni correction to protect against the risk of Type I errors caused by multiple comparisons has been criticized for causing a substantial reduction in the statistical power of rejecting an incorrect *H*_*o*_ in each test, i.e., increasing the risk of Type II errors [42]. Nevertheless, in our study the procedure seemed reasonable as the statistically significant results both appears to be clinically significant in terms of mean differences, and are supported by our results from an earlier qualitative study [7].

There were more female nurses than male (96/4%) participating in the study. Among the nurses who received the survey, 7% were male. According to the National Board of Health and Welfare [43], 12% of registered nurses in Sweden in 2015 were male, and 7% is likely to be a representative number for hematology and oncology wards, as more male nurses work in emergency, anesthetics/intensive care and psychiatry [44].

## Conclusions

Nurses and physicians need to talk openly about their different perspectives on DNR decisions, so they can develop a deeper understanding of the decisions, especially in cases where they disagree. They should also be aware that what they think is important is not always likely to happen. For nurses this is especially important, since physicians are the decision-makers, and nurses might avoid moral distress by knowing the reasons for DNR/CPR decisions. The organization needs to support these discussions through providing an environment that allows for ethical discussions on regular basis. Patients and relatives may also benefit from receiving the same information from all caregivers.

## Supporting information

S1 File Excel data set for SPSS. (XLSX)

## References

[1] Swedish Society of Medicine, Swedish Society of Nursing and Swedish Resuscitaion Council (Svenska Läkaresällskapet, Svensk Sjuksköterskeförening, and Svenska Rådet för Hjärt-lungräddning). Ethical Guidelines for CPR (Etiska riktlinjer för HLR). 2013 Apr 05. [cited 1 May 2018]. In Ethics in CPR [Internet]. Stockholm: Svenska Rådet för Hjärt-lungräddning. Available from: https://www.hlr.nu/wp-content/uploads/2018/03/Etiska-riktlinjer-för-HLR-Maj-2013.pdf.

[2] WHO. WHOQOL: Measuring Quality of Life 2018 [cited 15 August 2018]. In Health statistics and information systems [Internet]. Geneva: WHO Available from: http://www.who.int/healthinfo/survey/whoqol-qualityoflife/en/

[3] Swedish Resuscitation Council. Cardiac arrest in hospital–treatment and education (Hjärtstopp inom sjukvården—behandling och utbildning). 2017 Sep 27 [cited 30 April 2018]. In Cardiac arrest in hospital [Internet]. Stockholm: Svenska rådet för hjärt-lungräddning (Swedish Resuscitation Council). Available from: https://www.hlr.nu/wp-content/uploads/2018/03/Hjärtstopp-inom-sjukvården-2017.pdf

[4] SOSFS 2011:7. Lifesustaining treatment. Regulation and general advise. (Livsuppehållande behandling Föreskrifter och allmänna råd). 2011 Jul 6. [cited 1 May 2018]. In Regulation [Internet]. Stockholm: Socialstyrelsen (National Board of Health and Welfare). Available from: https://www.socialstyrelsen.se/Lists/Artikelkatalog/Attachments/18374/2011-6-26.pdf

[5] SFS 2014:821. Patient Act (Patientlag). 2014 Jun 19. [cited 1 May 2018]. In Document and Regulation [Internet]. Stockholm: Socialdepartementet (Social Department). Available from: https://www.riksdagen.se/sv/dokument-lagar/dokument/svensk-forfattningssamling/patientlag-2014821_sfs-2014-821

[6] Burns JP, Truog RD. The DNR Order after 40 Years. N Engl J Med. 2016 8 11 10.1056/NEJMp1605597 27509098 10.1056/NEJMp1605597

[7] Pettersson M, Hedstrom M, Hoglund AT. Striving for good nursing care: Nurses' experiences of do not resuscitate orders within oncology and hematology care. Nurs Ethics. 2014 6 9 10.1177/0969733014533238 24913543 10.1177/0969733014533238

[8] Silen M, Svantesson M, Ahlstrom G. Nurses' conceptions of decision making concerning life-sustaining treatment. Nurs Ethics. 2008 2 15 10.1177/0969733007086014 18272607 10.1177/0969733007086014

[9] Clements M, Fuld J, Fritz Z. Documentation of resuscitation decision-making: a survey of practice in the United Kingdom. Resuscitation. 2014 2 25 10.1016/j.resuscitation.2014.02.005 24560825 10.1016/j.resuscitation.2014.02.005

[10] Becerra M, Hurst SA, Junod Perron N, Cochet S, Elger BS. 'Do not attempt resuscitation' and 'cardiopulmonary resuscitation' in an inpatient setting: factors influencing physicians' decisions in Switzerland. Gerontology. 2011 11 26 10.1159/000319422 21099190 10.1159/000319422

[11] Olver I, Eliott JA. The perceptions of do-not-resuscitate policies of dying patients with cancer. Psychooncology. 2008 7 17 10.1002/pon.1246 17631674 10.1002/pon.1246

[12] Osinski A, Vreugdenhil G, de Koning J, van der Hoeven JG. Do-not-resuscitate orders in cancer patients: a review of literature. Support Care Cancer. 2017 2 10.1007/s00520-016-3459-9 27771786 10.1007/s00520-016-3459-9

[13] Levin T, Li Y, Weiner J, Lewis F, Bartell A, Piercy J. How do-not-resuscitate orders are utilized in cancer patients: Timing relative to death and communicating-training implications. Palliat Support Care. 2008 12 10.1017/S1478951508000540 19006588 10.1017/S1478951508000540

[14] Duplan KL, Pirret AM. Documentation of cardiopulmonary resuscitation decisions in a New Zealand hospital: A prospective observational study. Intensive Crit Care Nurs. 2016 12 10.1016/j.iccn.2016.06.005 27575617 10.1016/j.iccn.2016.06.005

[15] Olver IN, Eliott JA. Translating into practice cancer patients' views on do-not-resuscitate decision-making. Cancers (Basel). 2016 10 01 10.3390/cancers8100089 27690104 10.3390/cancers8100089 PMC5082379

[16] Brown M, Ruberu R, Thompson CH. Inadequate resuscitation documentation in older patients' clinical case notes. Intern Med J. 2014 1 24 10.1111/imj.12328 24450525 10.1111/imj.12328

[17] Jensen HI, Ammentorp J, Johannessen H, Ording H. Challenges in end-of-life decisions in the intensive care unit: an ethical perspective. J Bioeth Inq. 2013 3 10.1007/s11673-012-9416-5 23299401 10.1007/s11673-012-9416-5

[18] Mockford C, Fritz Z, George R, Court R, Grove A, Clarke B, et al Do not attempt cardiopulmonary resuscitation (DNACPR) orders: a systematic review of the barriers and facilitators of decision-making and implementation. Resuscitation. 2015 3 10.1016/j.resuscitation.2014.11.016 25433293 10.1016/j.resuscitation.2014.11.016

[19] Svantesson M, Sjökvist P, Thorsén H. End-of-life decisions in Swedish ICUs. Intensive Crit Care Nurs. 2003 8 10.1016/s0964-3397(03)00055-7 10.1016/s0964-3397(03)00055-7 12915113

[20] Meehan AD, Brosché L. A Vital Decision about Life–Doctors' and Nurses' Attitudes to Current Procedures for DNR-orders at Swedish University Hospital. Univers J Public Health, 2016; 4(2): 55–59.

[21] Pfeil TA, Laryionava K, Reiter-Theil S, Hiddemann W, Winkler EC. What keeps oncologists from addressing palliative care early on with incurable cancer patients? An active stance seems key. Oncologist. 2015 1 10.1634/theoncologist.2014-0031 25361623 10.1634/theoncologist.2014-0031 PMC4294613

[22] Mahon MM, McAuley WJ. Oncology nurses' personal understandings about palliative care. Oncol Nurs Forum. 2010 5 10.1188/10.ONF.E141-E150 20439199 10.1188/10.ONF.E141-E150

[23] Liao K, Blumenthal-Barby J, Sikora AG. Factors Influencing Head and Neck Surgical Oncologists' Transition from Curative to Palliative Treatment Goals. Otolaryngol Head Neck Surg. 2016 9 13 10.1177/0194599816667712 27625024 10.1177/0194599816667712

[24] Yuen JK, Reid MC, Fetters MD. Hospital do-not-resuscitate orders: why they have failed and how to fix them. J Gen Intern Med. 2011 7 10.1007/s11606-011-1632-x 21286839 10.1007/s11606-011-1632-x PMC3138592

[25] Udo C, Lovgren M, Lundquist G, Axelsson B. Palliative care physicians' experiences of end-of-life communication: A focus group study. Eur J Cancer Care (Engl). 2018 1 10.1111/ecc.12728 28727207 10.1111/ecc.12728

[26] Trivedi S. Physician perspectives on resuscitation status and DNR order in elderly cancer patients. Rep Pract Oncol Radiother. 2013 1 16 10.1016/j.rpor.2012.12.002 24381748 10.1016/j.rpor.2012.12.002 PMC3863213

[27] Pettersson M, Hedstrom M, Hoglund AT. Ethical competence in DNR decisions -a qualitative study of Swedish physicians and nurses working in hematology and oncology care. BMC Med Ethics. 2018 6 19 10.1186/s12910-018-0300-7 29914440 10.1186/s12910-018-0300-7 PMC6007064

[28] Polit DF, Beck CT. Nursing Research—Generating and assessing evidence for nursing practice. 8th ed Philadelphia: Lippincott Williams & Wilkins; 2008.

[29] SFS 2003:460. Act on Ethical Review of Researsh Involving Humans. (Lag om etikprövning av forskning som avser människor). 2003 May 05. [cited 1 May 2018]. In Document and Regulation [Internet]. Stockholm: Ministry of Education and Researsh (Utbildningsdepartementet). Available from: https://www.riksdagen.se/sv/dokument-lagar/dokument/svensk-forfattningssamling/lag-2003460-om-etikprovning-av-forskning-som_sfs-2003-460

[30] World Medical Association (WMA). The Declaration of Helsinki. Ethical Principles for Medical Research Involving Human Subjects. Fortaleza, Brazil WMA 2013. 10.1001/jama.2013.281053 24141714

[31] Jameton A. A reflection on moral distress in nursing together with a current application of the concept. J Bioeth Inq. 2013 10 10.1007/s11673-013-9466-3 24048753 10.1007/s11673-013-9466-3

[32] Young A, Froggatt K, Brearley SG. 'Powerlessness' or 'doing the right thing'—Moral distress among nursing home staff caring for residents at the end of life: An interpretive descriptive study. Palliat Med. 2017 10 10.1177/0269216316682894 28659023 10.1177/0269216316682894

[33] Prentice TM, Gillam L, Davis PG, Janvier A. Always a burden? Healthcare providers' perspectives on moral distress. Arch Dis Child Fetal Neonatal Ed. 2017 9 29 10.1136/archdischild-2017-313539 28970316 10.1136/archdischild-2017-313539

[34] Corley MC, Elswick RK, Gorman M, Clor T. Development and evaluation of a moral distress scale. J Adv Nurs, 2001 1 (33)2, 250–6. 11168709 10.1046/j.1365-2648.2001.01658.x

[35] Beckstrand RL, Collette J, Callister L, Luthy KE. Oncology Nurses' Obstacles and Supportive Behaviors in End-of-Life Care Providing Vital Family Care. Oncol Nurs Forum. 2012 9 10.1188/12.ONF.E398-E406 22940519 10.1188/12.ONF.E398-E406

[36] Swedish Society of Medicine (Svenska Läkaresällskapet). Hippocratic Oath. n.d; [cited 2 May 2018]. In Ethical codes [Internet]. Stockholm: Svenska Läkaresällskapet. Available from: http://www.sls.se/etik/etiska-koder/den-hippokratiska-eden/.

[37] Baruch Y, Holtom BC. Survey response rate levels and trends in organizational research. Human Relations. 2008 8 01 10.1177/0018726708094863.

[38] Cook DA, Wittich CM, Daniels WL, West CP, Harris AM, Beebe TJ. Incentive and Reminder Strategies to Improve Response Rate for Internet-Based Physician Surveys: A Randomized Experiment. J Med Internet Res. 2016 9 16 10.2196/jmir.6318 27637296 10.2196/jmir.6318 PMC5045523

[39] Cunningham CT, Quan H, Hemmelgarn B, Noseworthy T, Beck CA, Dixon E, et al Exploring physician specialist response rates to web-based surveys. BMC Med Res Methodol. 2015 4 09 10.1186/s12874-015-0016-z 25888346 10.1186/s12874-015-0016-z PMC4404667

[40] Ebert JF, Huibers L, Christensen B, Christensen MB. Paper- or Web-Based Questionnaire Invitations as a Method for Data Collection: Cross-Sectional Comparative Study of Differences in Response Rate, Completeness of Data, and Financial Cost. J Med Internet Res. 2018 1 23 10.2196/jmir.8353 29362206 10.2196/jmir.8353 PMC5801515

[41] Martins Y, Lederman RI, Lowenstein CL, Joffe S, Neville BA, Hastings BT, et al Increasing response rates from physicians in oncology research: a structured literature review and data from a recent physician survey. Br J Cancer. 2012 3 13 10.1038/bjc.2012.28 22374464 10.1038/bjc.2012.28 PMC3304407

[42] Nakagawa S. A farewell to Bonferroni: the problems of low statistical power and publication bias. Behavioral Ecology. 2004 11 10.1093/beheco/arh107

[43] Socialstyrelsen 2018. Statistik om legitimerad hälso-och sjukvårdspersonal 2016 samt arbetsmarknadsstatus 2015. (Statistics on Licensed Health Care Personnel (2016) and Workforce status (2015)). 2018 Feb 28. [cited 2 May 2018]. In Statistics per subject [Internet]. Stockholm: National Board of Health and Welfare. Available from: http://www.socialstyrelsen.se/publikationer2018/2018-2-9.

[44] Socialstyrelsen 2016. Tillgång på Specialistsjuksköterskor och röntgensjuksköterskor 2014. (Nurses in specialist training 1995–2014). 2016 Nov 03. [cited 2 May 2018]. In Statistics per subject [Internet]. Stockholm: National Board of Health and Welfare. Available from: http://www.socialstyrelsen.se/publikationer2016/2016-11-2.

